# White Matter Differences between Healthy Young ApoE4 Carriers and Non-Carriers Identified with Tractography and Support Vector Machines

**DOI:** 10.1371/journal.pone.0036024

**Published:** 2012-04-25

**Authors:** Laurence O'Dwyer, Franck Lamberton, Silke Matura, Monika Scheibe, Julia Miller, Dan Rujescu, David Prvulovic, Harald Hampel

**Affiliations:** 1 Department of Psychiatry, Psychosomatic Medicine and Psychotherapy, Goethe University, Frankfurt, Germany; 2 Centre for Imaging-Neurosciences and Applications to Pathologies, UMR 6232, CNRS, CEA, University of Caen and Paris Descartes, Caen, France; 3 Department of Psychiatry, Ludwig-Maximilians-University, Munich, Germany; 4 Department of Psychiatry, University of Halle-Wittenberg, Halle, Germany; University of Jaén, Spain

## Abstract

The apolipoprotein E4 (ApoE4) is an established risk factor for Alzheimer's disease (AD). Previous work has shown that this allele is associated with functional (fMRI) changes as well structural grey matter (GM) changes in healthy young, middle-aged and older subjects. Here, we assess the diffusion characteristics and the white matter (WM) tracts of healthy young (20–38 years) ApoE4 carriers and non-carriers. No significant differences in diffusion indices were found between young carriers (ApoE4+) and non-carriers (ApoE4−). There were also no significant differences between the groups in terms of normalised GM or WM volume. A feature selection algorithm (ReliefF) was used to select the most salient voxels from the diffusion data for subsequent classification with support vector machines (SVMs). SVMs were capable of classifying ApoE4 carrier and non-carrier groups with an extremely high level of accuracy. The top 500 voxels selected by ReliefF were then used as seeds for tractography which identified a WM network that included regions of the parietal lobe, the cingulum bundle and the dorsolateral frontal lobe. There was a non-significant decrease in volume of this WM network in the ApoE4 carrier group. Our results indicate that there are subtle WM differences between healthy young ApoE4 carriers and non-carriers and that the WM network identified may be particularly vulnerable to further degeneration in ApoE4 carriers as they enter middle and old age.

## Introduction

Apolipoprotein E (ApoE) regulates the metabolism of lipids by coordinating their transport and redistribution from one cell type to another via ApoE receptors and proteins associated with lipid transfer and lipolysis [1]. ApoE is thought to play a key role in neuronal development and brain plasticity [2]. There are three allelic variants of the ApoE gene in humans (E2, E3, E4) [3]. Although the frequency of the ApoE4 allele is low in humans, primate studies suggest that it is the ancestral allele and it has been hypothesized that the mutation leading to ApoE3 may have been selected for during the evolution of the unique role of grandmothering in early humans which led to the spread of the E3 allele [3].

The E4 allele has been shown to confer a higher risk of developing both early onset and late onset AD [4], [5]. Brain structure and function have been found to be altered in ApoE4 carriers, both in AD patients [6]–[9] and in healthy subjects [10]–[14]. Studies have found greater rates of temporal lobe atrophy in AD patients with greater load of E4 allele [6], [15]–[17] and reduced medial temporal lobe volumes in healthy ApoE4 carriers across the age spectrum [14], [18]–[21]. However, a number of studies have also failed to replicate these findings [22]–[24]. A mixed picture is also found in terms of functional studies, with both increased [6], [9], [25] and decreased [26], [27] task-related BOLD signals reported in carrier groups relative to non-carriers.

As the E4 allele has been related to several deleterious biological effects, the question arises as to why it has persisted. Recent work has suggested that the ApoE4 genotype might exert an antagonistic pleiotropic effect [28] whereby carriers have contrasting effects across the lifespan, with significant negative effects occurring in middle to old age, but with specific advantages noted in healthy young carriers. This pleiotrophic effect of E4 may account for some of the discrepancies noted in the studies above. E4 has also been associated with higher IQ [29], a higher education level [30], reduced cardiovascular response to experimentally induced stress [31], a protective effect against spontaneous abortion during embryogenesis [32], and against perinatal death [33]. Additionally, hippocampal LTP was enhanced at a young age in knock-in mice expressing human APOE4 [34].

While the majority of studies to date have focussed on GM changes, few studies have investigated the WM characteristics of ApoE4 carriers and non-carriers. As the ApoE gene is involved in the transport of lipids which in turn are used for the construction of myelin sheaths [35], [36], the role of WM development in ApoE4 carriers and non-carriers will clearly be of great importance. In Alzheimer disease (AD) WM pathology has been shown to correlate with disease severity [37]–[39]. WM changes may also be a key indicator of early pathology [40] and WM damage in AD has been highlighted both in postmortem studies [41] as well as in *in vivo* MRI studies [42]–[44]. Degradation of WM microstructure can occur secondary to grey matter (GM) pathology via an accumulation of aggregated hyperphosphorylated tau protein and the deposition of Aβ [45]. There is also evidence for direct WM damage occurring as a result of oligodendrocyte death and reactive gliosis [46].

To date two previous DTI studies in healthy older people found subtle but significant decreases in FA in the parahippocampal gyrus [47] and in the splenium of the corpus callosum [48] in ApoE4 carriers. A recent whole brain tract based spatial statistics (TBSS) study found widespread significant differences between healthy young carriers and non-carriers [49]. Therefore, the few studies that exist indicate the possibility that ApoE4 carriers exhibit WM damage in early adulthood.

The aim of the current study was to investigate the effect of APOE genotype on WM structure in healthy young people. Whole brain TBSS was applied to detect significant differences that may be present between the groups. Diffusion data derived from TBSS was also run through a feature selection algorithm (ReliefF) [50] which identified the most salient voxels for group classification that were subsequently employed in the training of a support vector machine (SVM) classifier. The SVM was then used to classify the two groups. The voxels selected by the ReliefF algorithm were also used as seeds for probabilistic tractography.

We hypothesized that tractography initiated from seed voxels selected by ReliefF would identify a WM network that may be structurally different between carrier and non-carrier groups. We also hypothesized that the SVM would be able to classify the two groups using diffusion information from voxels selected by the ReliefF algorithm.

## Methods

### Ethics Statement

The study was approved by the Ethics Committee of Goethe University and was in accordance with the Declaration of Helsinki. All participants provided informed written consent.

### Participants

44 cognitively intact persons between 20 and 38 years of age, all without any history of neurological or psychiatric disease were assessed in the current study. These 44 subjects were drawn from a larger cohort of 96 subjects. All of the 44 selected subjects were right-handed, as assessed with the Edinburgh Handedness Inventory [51] and provided written informed consent. Ethics approval was obtained from the local ethics committee of JWG University Frankfurt. All subjects underwent neuropsychological assessment. Verbal learning and memory was assessed using the German Version of the California Verbal Learning Test (CVLT) [52], [53], visual memory was tested with the Brief Visual Memory Test - R (BVMT R) [54]. Additionally, measures of working memory and attention were obtained using the Letter Number Sequencing (LNS) [55], Spatial Span of the Wechsler Memory Scale 3 (WMS SS) [56] and Trail Making Test A (TMT). The verbal IQ was tested with a German verbal intelligence test (Mehrfachwahl-Wortschatz-Test B; MWTB), in which subjects had to indicate real words within lists of pseudo-words [57]. Depressive Symptoms were measured with the German Version of the Beck Depression Inventory (BDI 2) [58], [59].

All participants from the larger cohort (n = 96) underwent APOE genotyping using PCR and sequencing. For the current analysis, 21 subjects who were heterozygote for ApoE4 (ε3/ε4) and one subject who was homozygote for ApoE4 were included into the ε4+ group. 22 subjects, matched for age, gender and education who were ε4 negative (ε3/ε3) were included into the ε4 group. Group characteristics are summarized in Table 1.

**Table 1:** Demographic and cognitive characteristics of the sample groups.

	APOE4 non-carriers		APOE4 carriers			
Variable	Mean	SD	Mean	SD	T-value	P-value
	n = 22			n = 22		
Age (years)	26.7	4.0	26.9	5.3	5.28	0.92
Gender (m/f)	13/9			13/9		0.76
Education (years)	16.8	4.5	17.0	4.3	−0.15	0.88
MWTB	29.7	3.6	30.3	4.3	−0.46	0.65
MWTB IQ	106.7	23.6	114.7	15.3	−1.28	0.21
TMT (sec)	22.0	5.8	19.3	3.9	1.81	0.08
WMS SS	19.1	2.0	19.4	2.8	−0.31	0.76
LNS	18.7	3.2	17.8	2.7	1.07	0.29
BVMT R	32.7	3.5	32.0	3.6	0.62	0.54
BDI 2	3.2	3.6	2.4	2.9	0.83	0.41
CVLT	66.8	8.0	64.5	9.1	0.90	0.37

Values are mean ± standard deviation. Significance was set at p<0.05; thus no significant differences were found between the groups. Values denote mean and standard deviation or number of subjects. P-values refer to t-tests (parametric tests) and chi-square tests (for categorial data). Abbreviations: MWTB: Mehrfachwahl-Wortschatz-Test B, a German Verbal intelligence test; TMT: trail making test; WMS SS: Spatial Span of the Wechsler Memory Scale; LNS: Letter Number Sequencing; BVMT R: Brief Visual Memory Test R; BDI 2: Beck Depression Inventory 2; CVLT: California Verbal Learning Test.

### ApoE4 Genotyping

APOE genotyping of the two determinating variants rs7412 and rs429358 was analyzed using pre-designed TaqMan SNP Genotyping assays (Applied Biosystems, Foster City, CA). Briefly for each SNP 20 µl reaction mix contained 15 ng genomic DNA, unlabeled PCR primers, MGB labeled probes (VIC, 6FAM), 10 µl of 2× TaqMan universal PCR Master Mix (Applied Biosystems, Foster City, CA). PCR was performed on an ABI 7000 instrument (Applied Biosystems, Foster City, CA) with the following cycling programm: 95°C for 15 s, 40 cycles of 95°C for 15 s and 60°C for 60 s. The ABI 7000 genotyping software was used for allelic discrimination.

### Imaging Methods

All MR images were acquired using a Trio 3-T scanner (Siemens, Erlangen, Germany) with a standard head coil for radiofrequency transmission and signal reception. Participants were outfitted with protective earplugs to reduce scanner noise and a hand-held response device. For T1 weighted structural brain imaging, an optimized 3D modified driven equilibrium Fourier transform (3D MDEFT) sequence was used with the following parameters: acquisition matrix = 256×256, repetition time (TR) = 7.92 ms, echo time (TE) = 2.48 ms, field of view = 256 mm, 176 slices, 1.0 mm slice thickness.

DTI was acquired using an echo planar imaging (EPI) sequence with the following pulse sequence: TR = 8760 ms; TE = 100 ms; acquisition voxel size = 2×2×2 mm^3^; 60 axial adjacent slices; slice thickness = 2 mm (no gap); FOV = 192 mm×192 mm×120 mm; acquisition matrix = 96×96; 10 images without diffusion weighting and 60 diffusion-encoding gradients applied in 60 noncollinear directions; b-value = 1000 s/mm^2^; both the b0 and the 60 diffusion weighted images were averaged three times, bandwidth = 1302 Hz/pixel; total acquisition time = 10 min 31 sec. This sequence was acquired using generalised auto-calibrating parallel acquisitions (GRAPPA; Griswold et al., 2002) with an acceleration factor of 2.

For each subject a total of three consecutive DTI scans were acquired. An average of these three scans was then created for use in subsequent DTI processing.

A T2-weighted fluid attenuation inversion recovery (FLAIR) sequence was also acquired to ensure that vascular pathology was not significant. For all 44 subjects selected from the larger cohort, no hyperintense white matter lesions were seen in the FLAIR scans.

### High Resolution T1W Structural Image Processing

Images were skull stripped with the Brain Extraction Tool (BET) from the FSL library [60]. Brain tissue volume, normalized for subject head size, was estimated with SIENAX [61], [62], which is part of the FSL library. SIENAX starts by extracting brain and skull images from the single whole-head input data. The brain image is then affine-registered to MNI152 space [63], [64] (using the skull image to determine the registration scaling); this is primarily in order to obtain the volumetric scaling factor, to be used as a normalization for head size. Next, tissue-type segmentation with partial volume estimation is carried out [65] in order to calculate total volume of brain tissue including separate estimates of volumes of WM and GM.

### DTI Processing

DTI analysis was performed using TBSS [66]. Images were skull stripped with the Brain Extraction Tool (BET) from the FSL library [60]. Raw DTI images were first corrected for motion and eddy current effects. The diffusion tensor was then calculated with the DTIFIT program for whole brain volumes and the resulting FA maps, together with the DA (λ1) and DR ((λ2+λ3)/2) and MD ((λ1+λ2+λ3)/3) maps, were used in subsequent TBSS analysis.

TBSS performs a non-linear registration that aligns each FA image to every other one and calculates the amount of warping needed for the images to be aligned. The most representative image is determined as the one needing the least warping for all other images to align to it. The FSL library also provides a 1 mm isotropic FA target image (FMRIB58_FA) in standard space, which is sometimes used instead of the most representative image from the study cohort. This can be problematic as the target image is based on a young healthy brain. Using the method of “all subject to all subject” registration is more computationally intensively, but highly desirable when dealing with populations other than young healthy controls. After this registration step, warped versions of each subject's FA image were generated which were then averaged and a white matter “skeleton” was then created suppressing all non-maximum FA values in each voxel's local-perpendicular direction and subsequently comparing all remaining non-zero voxels with their nearest neighbours, thus searching for the centre of fibre bundles. The skeleton was then thresholded at an FA value of 0.2 which limits the effects of poor alignment across subjects and ensures that GM and CSF voxels are excluded from the skeleton. The resulting skeleton contained WM tracts common to all subjects. A “distance map” is then created which is used to project each FA image onto the mean FA skeleton that is common to all subjects [66]. The same non-linear transformations derived for the FA maps were applied to the DA, DR and MD maps.

Following TBSS processing, a global region of interest was created using the white matter skeleton that is common to all subjects. Mean values of FA, DA, DR and MD were extracted from each subject using this global ROI.

For statistical analysis, the images were analyzed using the “randomise” tool from FSL using a standard GLM design which controlled for the effect of age and gender. Randomise computes permutation tests on the assumption that the null hypothesis implies complete exchangeability of the observations [67]. Using this setup voxelwise differences between groups were then assessed, setting the number of permutations at 5000 permutations. Significance was tested at p<0.05 levels, corrected for multiple comparisons using the “2D” parameter settings with threshold-free cluster enhancement (TFCE), a method which avoids using an arbitrary threshold for the initial cluster-formation [68].

### Support Vector Machines Analysis

Classification of individual subjects was undertaken using the freely available WEKA software package (http://www.cs.waikato.ac.nz/ml/weka, Version 3.6.4) [69], [70]. Following TBSS analysis, the skeletonised FA, DA, DR and MD data was analysed in Matlab (program written by FL and available on request), which extracted the diffusion values from the WM skeleton. There were 132,208 voxels in the WM skeleton and diffusion values for all indices of diffusion were extracted from each voxel in the WM skeleton. Classification between carrier and non-carrier groups was undertaken using each index of diffusion separately in order to determine the most efficient index for classification.

The first step of the WEKA analysis was to reduce the number of voxels to those that are most relevant for classification. This step eliminates non-discriminative voxels which would reduce classification accuracy. The feature selection algorithm “ReliefF” was used to extract the most important voxels [50]. For each classification group and also for each index of diffusion, eight reduced datasets were created as follows:

1. 250 voxel dataset
2. 500 voxel dataset
3. 750 voxel dataset
4. 1000 voxel dataset
5. 2000 voxel dataset
6. 3000 voxel dataset
7. 4000 voxel dataset
8. 5000 voxel dataset

Therefore in total, 32 reduced datasets were created; i.e. 8 reduced datasets for each index of diffusion (FA, MD, DR and DA). The choice of identifying these 8 datasets is based on previous studies that have shown that this is an optimum range of data reduction for successful classification [71]–[74].

The aim of the ReliefF algorithm is to estimate the quality of voxels according to how well the value of a voxel distinguishes between instances that are near to each other. The central idea of ReliefF is to estimate the quality of voxels according to how well their values distinguish between instances that are near to each other [50], [75]. Given a randomly selected instance *Ins_m_* from class *L*, ReliefF searches for *K* of its nearest neighbours from the same class called nearest hits *H*, and also *K* of its nearest neighours from each of the different classes, called nearest misses *M*. It then updates the quality estimate *Wi* for voxel *i* based on the values for *Ins_m_*, *H* and *M*. If instance *Ins_m_* and those in *H* have different values for voxel *i*, then the quality estimation *Wi* is decreased. Alternatively, if instance *Ins_m_* and those in *M* have different values on the voxel *i*, then *Wi* is increased [50], [75].

After reducing the data into the three datasets of differing sizes, classification was then performed using the SVM algorithm “sequential minimal optimization” (SMO) [76] with a radial basis function (RBF) kernel [77]. SVMs are algorithms that learn how to assign labels to objects [78]. They use linear models to implement nonlinear class boundaries by transforming the input into a new higher dimensional space. In this way, a straight line in the new space can be curved or non-linear when transformed back to the original lower-dimensional space. Following transformation, a linear model called the maximum margin hyperplane is created. To visualise this, imagine a dataset with two-classes that are linearly separable. The maximum margin hyperplane is the one that gives the greatest separation between the classes. The hyperplane describes a straight line in a high-dimensional space, and therefore a separating hyperplane is a line that separates the classes. The instances that are closest to the maximum margin hyperplane are called support vectors. A unique set of support vectors defines the maximum margin hyperplane for the learning problem. Once the support vectors for the two classes are established, a maximum margin hyperplane can be constructed.

The projection of the data from low dimensional space to higher dimensional space is achieved with a kernel function. The optimal kernel function is usually found by trial and error [78]. In the current study a radial basis function (RBF) kernel was used to nonlinearly map samples into a higher dimensional space. RBF kernels use two parameters: C and GAMMA. GAMMA represents the width of the radial basis function, and C represents the error/trade-off parameter that adjusts the importance of the separation error in the creation of the separation surface [79]. C was fixed to 1 and GAMMA was fixed to 0.01. During each trial, tenfold cross validation was used for classification. This involves dividing the data into 10 parts. Nine parts were used for training and one part for testing. This was repeated 10 times, resulting in the learning algorithm being implemented 100 times on datasets that are all nine-tenths the size of the original [69], [70]. This is a standard procedure in machine learning which reduces the variation related to data selection and allows results to be averaged to yield robust calculations of the performance of the SVM. During each of the ten repetitions within a given trial, the same values of C and GAMMA are retained for each repetition.

For the analysis of results, measures of sensitivity, specificity and an F-measure were used. An F-measure is used to give a single measure that can characterise overall performance [70]. An F-measure is the harmonic mean of Precision and Recall.

**Figure 1:**
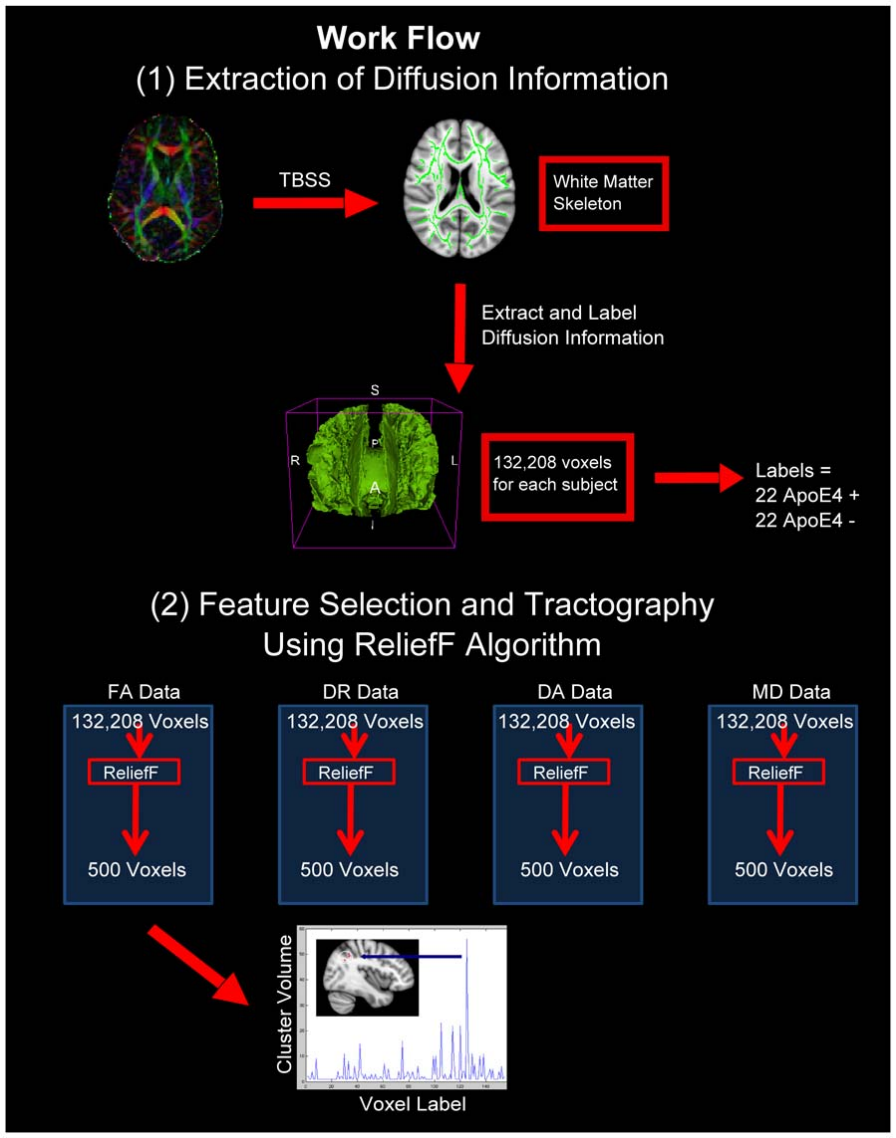
Workflow for the processing of white matter diffusion information. (1) Following TBSS processing a white matter skeleton is created which is common to all subjects. Diffusion values are then extracted from every voxel in the white matter skeleton using a custom made matlab script. Data is then labelled as either ApoE4 carrier or non-carrier. (2) Following preparation of the data into a WEKA compatible format the data is reduced using a feature selection algorithm, ReliefF. This algorithm selects the most salient voxels for group classification. These voxels are then used for training a support vector machine (SVM) classifier and subsequently the classification is performed with this classifier. The lowermost figure shows a readout of top 500 voxels selected from the full FA dataset. The y-axis represents the volume of particular clusters and the location of the largest cluster is shown circled in white in the anatomical images.

A workflow, outlining the creation of the WM skeleton, the extraction of diffusion values from each point on the WM skeleton, the reduction of the data using ReliefF and the application of the SVM is shown Fig. 1.

### Tractography

Seeds were created from the significant clusters created from the ReliefF algorithm, and these seeds were used for probabilistic tractography [80]. In order to perform the probabilistic tractography in standard space for each subject, we fed both warpfields generated with FNIRT when registering individual subjects to standard space, and their corresponding inversed warpfields into the tractographic algorithm. A multi-fibre model was fit to diffusion data at each voxel which allows tracing of fibres through regions of fibre crossing and complexity [81]. At each voxel, a probability distribution function (pdf) is estimated on each fibre direction. Tractography then proceeds by drawing multiple (in this case 5,000) streamline samples through these pdfs from each seed voxel to create an estimate of the distribution of connections from each seed voxel. When these streamlines reach a voxel in which more than one direction is estimated they follow the direction that is closest to parallel with the direction at which the streamline arrives. Generated pathways are volumes in which values at each voxel represent the number of samples passing through that voxel and, therefore, the probability of connection to the seed voxel. To remove spurious connections, pathways in individual subjects were thresholded to include only voxels which had at least 250 samples passing through them (out of 5,000) generated from each seed voxel. These pathways were then binarised and overlaid to create population probability maps in which voxel values represent the number of subjects in whom a pathway is present. As is noted in the Results and Discussion sections, tractography was performed using the seed points from the FA dataset because this diffusion index produced the greatest classification accuracy.

## Results

### Demographic and Cognitive Characteristics

There were no significant differences between the groups in terms of any of the demographic or psychological measures taken (Table 1).

### Normalised Grey Matter and White Matter Volumes

**Figure 2:**
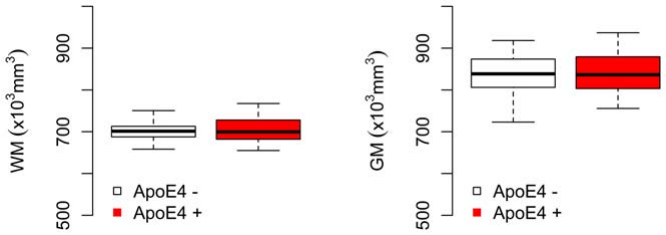
Normalised WM (left) and GM (right) volumes for healthy young ApoE4 carrier and non-carrier groups. No significant differences were found between the groups for either WM or GM volumes.

Normalised Grey Matter and White Matter Volumes were not significantly different between groups (p>0.05) (Fig. 2).

### Differences in Multiple Indices of Diffusion between ApoE4 Carriers and Non-Carriers

**Figure 3:**
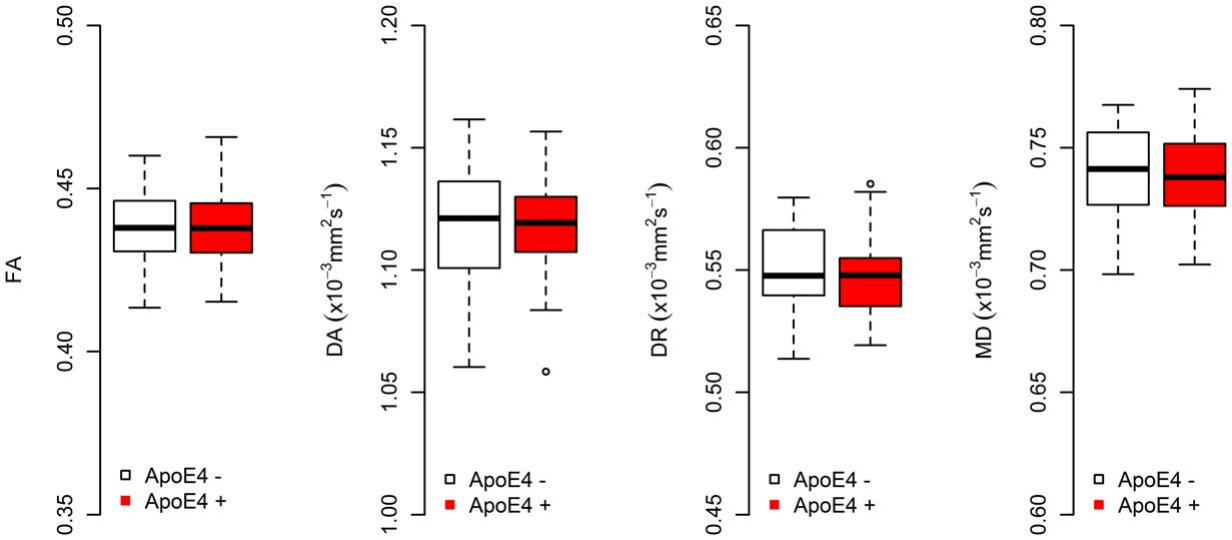
Boxplots showing the distribution of diffusion tensor MRI indices for the global WM skeleton in ApoE4 carriers (red) and non-carriers (white). The boxplots represent the interquartile ranges, which contain 50% of individual subjects' values. The whiskers are lines that extend from the box to the highest and lowest values. No significant differences were found between the groups.

After correcting for multiple comparisons no significant voxelwise differences were found between the two groups using randomise permutation testing of the TBSS images. Global boxplots for the four indices of diffusion can be seen in Figure 3. The diffusion values in these boxplots are taken from the entire white matter skeleton.

### SVM Classification of ApoE4 Carrier and Non-Carrier

**Figure 4:**
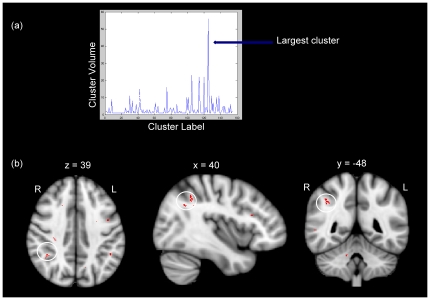
Voxels selected by the ReliefF algorithm as the most salient for group classification. (a) A graph of the most significant clusters of voxels selected by the ReliefF algorithm. This graph shows the cluster label on the x-axis and the volume of the contiguous clusters on the y-axis. (b) A selection of the top 500 voxels identified by the ReliefF algorithm. The white circles highlight the location of the largest cluster as identified by the previous graph. Note that this figure highlights the reduction of the entire FA dataset to the top 500 voxels. This process is followed for the top 250, 500, 750, 1000, 2000, 3000, 4000 and 5000 voxels of the FA dataset. This entire procedure is also repeated for DA, MD and DR datasets. The coordinates shown for each slice are MNI coordinates in millimetres. The labels L and R indicate left and right respectively. The middle panel (x = 40) indicates a sagittal slice in the right hemisphere.

The ReliefF algorithm was used to reduce the FA, DA, DR and MD datasets to datasets that contain the most salient voxels for group classification. Figure 4(a) presents a graphical depiction of the reduction of the FA dataset by ReliefF to the 500 most import voxels as judged by the weighting procedure described in the methods. The volume of clusters is noted on the y-axis. Figure 4(b) depicts the anatomical location of some of the voxels selected by ReliefF following the reduction of the FA dataset. The largest cluster of voxels is also indicated in the figure. The same process was followed when reducing the FA data to 250, 750, 1000, 2000, 3000, 4000 and 5000 voxel datasets. The entire procedure was repeated for the DR, MD and DA datasets.

**Figure 5:**
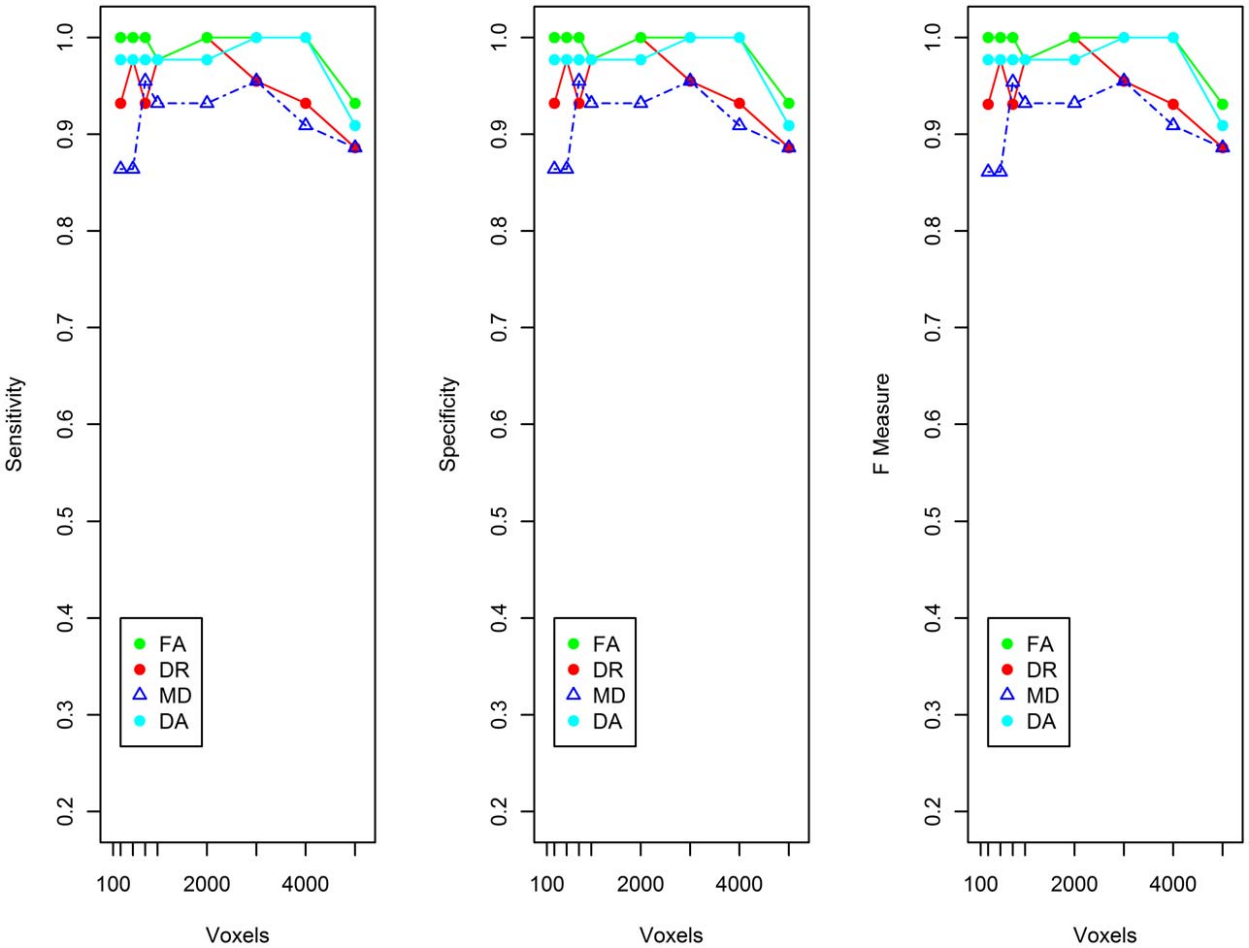
Sensitivity, Specificity and F-measure for SVM run on ApoE4+ and ApoE4− subjects. The F-measure is the harmonic mean of precision and sensitivity and can be used as a single measure of performance of the classification.

The reduced datasets were then used by the SVM for training and subsequent classification of ApoE4 carriers and non-carriers. An SVM analysis was performed separately using FA, DA, DR and MD datasets. The results from these four different SVM analyses are shown in Figure 5. The FA data produced the best overall performance, with sensitivity and specificity ranging from 93 to 100%. For the DA, DR and MD indices of diffusion, classification performance was slightly lower, with sensitivity and specificity in the range of 86–100% (Fig. 5).

### Tractography

**Figure 6:**
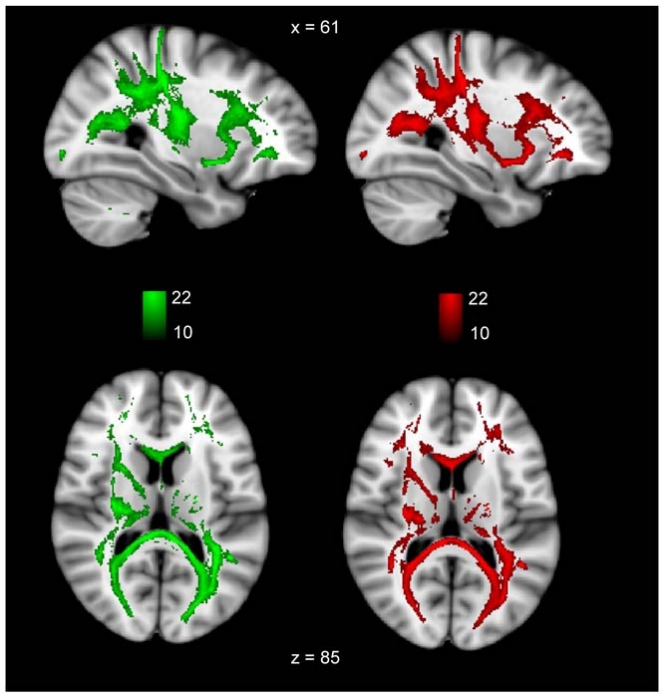
Tractography using seed points selected by the ReliefF algorithm for ApoE4 carriers and non-carriers, after reducing the full FA dataframe. Here we have heatmaps created from tracts that were thresholded at 250 out of 5,000 streamlines. At one end of the scale, the bright green (ApoE4 non-carrier) or bright red (ApoE4 carrier) indicates that tracts were present in all 22 subjects in each group, whereas at the other end, the dark green or dark blue indicates that tracts were present in 10 out of the total number of 22 subjects in each group.

**Figure 7:**
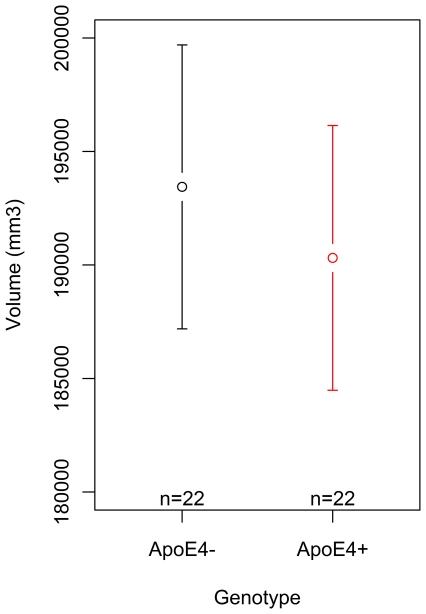
Volume of tracts produced using a seed of the top 500 voxels created by the ReliefF algorithm from the full FA dataset. The tracts were thresholded to include only WM tracts that contained 250 or more streamlines, out of a total of 5000 streamlines. ApoE4 carriers exhibited a non-significant (p = 0.45, t = 0.76) decrease in tract volume relative to non-carriers (Fig. 7). Mean tract volume was 190311.3 (SD 13151.6) mm^3^ for ApoE4 carriers and 193441.9 (SD 14105.5) mm^3^ for ApoE4 non-carriers.

As the voxels selected by ReliefF from the FA dataset produced the best overall performance in terms of group classification, the top 500 voxels from the FA data were used as seed points for tractography. Thus, tracts were initiated from all 500 of these voxels using probabilistic tractography in each ApoE4 carrier and non-carrier subject.These tracts (binarised at a threshold of 250 out of 5000 streamlines) are overlaid to create population probability heatmaps in which each voxel value represents the number of subjects in whom a pathway is present (Fig. 6). These population probability heatmaps highlight the WM network that is initiated from the 500 voxel seed created with the ReliefF algorithm. As these tracts were initiated from seed points that were chosen by ReliefF as being the most useful for locating differences between the groups, these heatmaps give an indication of tracts that may be susceptible to micro and macrostructural damage in ApoE4 carriers. The WM network implicated in both carrier and non-carrier groups extends into much of the parietal lobe, including the posterior cingulum and precuneus. The forceps major, forceps minor, superior longitudinal fasciculus, as well as the uncinate fasciculus and large portions of the prefrontal cortex are also implicated. Finally, an analysis of the total volume of tracts created from this 500 voxel seed is shown in Figure 7. ApoE4 carriers exhibited a non-significant (p = 0.45, t = 0.76) decrease in tract volume relative to non-carriers (Fig. 7). Mean tract volume was 190311.3 (SD 13151.6) mm^3^ for ApoE4 carriers and 193441.9 (SD 14105.5) mm^3^ for ApoE4 non-carriers.

## Discussion

The current results show that it is possible to classify healthy young ApoE4 carriers and non-carriers using an automated procedure based on structural WM differences between the two groups. Previous work has distinguished between healthy older subjects and subjects with mild cognitive impairment (MCI) using a combination of DTI and SVMs [74] but to the best of our knowledge no machine learning paradigm has attempted to classify healthy young ApoE4 carriers and non-carriers. Using diffusion data in combination with an SVM, our machine learning approach was able to classify ApoE4 carrier and non-carrier groups with an extremely high degree of accuracy.

Of note is the fact that the FA index produced the best classification performance. This is in line with a previous SVM classification study which also found that FA gave the greatest accuracy for the classification of healthy control and MCI subjects [74]. The reduced FA dataset was studied in further detail to assess the locations of the voxels selected by the ReliefF algorithm. This feature selection algorithm is able to effectively provide quality estimates for each voxel in the white matter skeleton. It has previously been used successfully in a number of different domains including genetics [82] and proteomics [83], and has recently begun to be used in MRI [74]. In the current study we see that it filters out irrelevant voxels and selects the most useful voxels which can be used by an SVM for training and classification. The largest cluster identified by ReliefF from the FA dataset was found in the right anterior parietal region. Interestingly, this region has been shown to demonstrate increased BOLD response in older ApoE4 carriers relative to non-carriers during picture learning [13].

It has been suggested, on the basis of functional changes in ApoE4 carriers, that the E4 allele modulates neuronal activity decades before any expression of disease [8], [84]. A significant interaction between age and E4 status has also been noted in the frontal lobe and other areas, such that aging was associated with decreased activity in E4 carriers and increased activity in non-carriers [85]. Therefore the ApoE genotype determines age-related changes in brain function that may reflect the increased vulnerability to cognitive decline in later life. Our finding of a subtle reduction in tract volumes in carriers may also be an indicator of an increased vulnerability of ApoE4 carriers in the tracts identified by tractography.

Also of note is the fact that 63% of the voxels (315 out of 500 voxels) selected by ReliefF were located in the right hemisphere. Laterality effects have been noted in a number of ApoE4 studies and evidence suggests that frontal-executive processes may be responsible for mediating a right hemisphere compensation in older age [86] [10] [87]. Bondi and colleagues found that the anterior cingulate was directly implicated in a right hemisphere compensatory mechanism response in healthy older ApoeE4 carriers [87]. Laterality effects have also been noted in older ApoE4 carriers during an object naming task, where carriers were found to have greater right perisylvian activation [88]. Overall, evidence suggests that the right hemisphere may be activated to a heightened degree in older ApoE4 carriers in order to compensate for early declines in episodic memory. Although the underlying reasons for this laterality are not known it is of interest that a model of aging, termed HAROLD (hemisphere asymmetry reductions in older adults) [89] posits that there is an age-related loss of hemispheric asymmetry of task-related activation in the prefrontal cortex (PFC). While healthy young subjects show greater activation in left PFC compared with the right PFC during the execution of a variety of cognitive tasks, many studies indicate that this laterality is reduced in older subjects [89]–[93]. This loss of laterality is frequently related to increased right PFC activation in older adults which has been suggested to serve as a possible compensatory mechanism in order to maintain cognitive performance in older age. The large number of functional activation studies showing increased right hemisphere functional activation in older age [89]–[93] may serve as indirect evidence for chronically stressed right hemisphere structures being predilection sites for structural alterations in neurodegenerative diseases such as AD. Subjects at genetic risk of AD (e.g. ApoE4 carriers) may display an increased vulnerability of these same predeliction sites to structural damage even at younger ages. A number of recent MCI and AD dementia classification studies have also noted a right-more-than-left pattern WM damage [94]–[96]. It is tempting to consider that ReliefF's identification of a large number of clusters in the right hemisphere, may represent key WM nodes in ApoE4 carriers which may be particularly sensitive to WM damage in later life when the negative effects of the ApoE4 gene become more pronounced.

The WM tracts initiated from the 500 voxels selected by ReliefF include significant portions of the parietal lobe, the cingulum, the dorsolateral prefrontal cortex, forceps major, forceps minor and the uncinate fasciculus. It is of interest that the posterior parietal lobe including the posterior cingulum/precuneus is part of the network identified, as this is also an important hub which sustains information transfer between the parahippocampal gyrus and the prefrontal cortex [94], [97]. A number of studies have proposed that damage to the posterior cingulum/precuneus leads to dysfunction of a network that is responsible for sustaining memory function [42], [94], [97]–[99]. The precuneus is connected to the temporal lobe via the retrosplenial cortex, and the hippocampus may not be able to communicate with the neocortex if it is sufficiently damaged [94], [97], [98]. PET studies have also highlighted reduced metabolism in the posterior cingulum and the prefrontal cortex in both MCI and AD subjects [100], [101].

Analysis of the total volume of tracts initiated from the 500 voxel seed indicates a non-significant decrease in volume in the ApoE4 carrier group. Overall, these results suggest that there are subtle structural WM differences between ApoE4 carriers and non-carriers. When considered together with evidence from previous studies, it is possible that while the structural differences are subtle at a young age, the WM network that has been highlighted by tractography may be more susceptible to further structural damage in ApoE4 carriers as they progress into middle and old age.

It is of particular interest that although no significant differences were found between the groups for any index of diffusion when inspecting TBSS results that are corrected for multiple comparisons, the voxels selected by ReliefF could still be employed by an SVM for successful classification. The reason for this lies in methodological differences between the two approaches. For TBSS, each voxel is assigned a t-value that is corrected for multiple comparisons, taking into account the fact that there are a total of 132,208 voxels in the entire white matter skeleton. The level of correction for multiple comparisons is therefore extremely high due to the large number of total voxels to be considered. However, the SVM approach is underpinned by a different theoretical framework. First, the most salient voxels are identified using the ReliefF algorithm. After this data reduction step the majority of voxels in the WM skeleton are discarded as irrelevant for subsequent SVM classification. Therefore, the SVM only considers the voxels identified by ReliefF in its calculation of a single parameter per subject, and does not run into the problem of multiple comparison biases [74].

It should be stressed that possession of the ApoE4 allele does not automatically imply the possibility of cognitive or structural deficits within the carrier group. A growing body of evidence suggests that the ApoE4 genotype may confer some beneficial effects at a young age, while being detrimental in older age [29]–[34]. Thus, there may be evolutionary reasons for the retention of the ApoE4 genotype because of its possible beneficial effects at a young age, which is offset by an increased risk of cognitive deficits in older age. Results from the current study however suggest that the ApoE4 gene may have no beneficial effects on WM structure in healthy young people.

On the basis of the current results, subtle microstructural WM differences do appear to be present in healthy young carrier subjects, which can be utilised by SVMs for group classification. The approach outlined in this study may offer a way to study a particular WM network longitudinally, based on the hypothesis that the network identified will be preferentially susceptible to structural damage in ApoE4 carriers in later life.

Only one previous study has examined structural WM differences between healthy young carrier and non-carrier groups [49]. The current results which found no significant corrected TBSS differences between the groups are at odds with this previous study which found significant FA decreases in young carriers relative to non-carriers. In fact, the FA reductions in young carriers relative to non-carriers found by Heise and colleagues appeared to be as widespread as the FA reductions noted previously in Alzheimer's patients relative to healthy older controls [42], [43], [98]. The age, sample size and overall demographics of the current cohort are comparable to those of Heise and colleagues; therefore, it might be possible that larger sample sizes need to be employed in future studies in order to determine whether or not widespread FA reductions in healthy young ApoE4 carriers relative to non-carriers can be replicated in the future.

To the best of our knowledge this is the first attempt to classify healthy young ApoE4 carrier and non-carrier groups with machine learning techniques. The use of 10 times 10-fold cross-validation [70] ensures the robustness and generality of the results by running the learning algorithm 100 times during each experiment. This method also reduces the effect of random variation when different folds are selected [69]. The use of a joint TBSS/SVM analysis allows information to be used from the entire brain which is a significant advantage over ROI based approaches for classification. The current methodology also obviates the need for the labour intensive selection and creation of ROIs.

Some limitations of the study should be noted. While the TBSS approach strives to avoid the problems of voxel based morphology relating to partial voluming, small WM tracts may still be contaminated with GM if the tract width is smaller than the original voxel size [66]. However, applying an FA threshold of 0.2 or less to the WM skeleton in the TBSS preprocessing steps is thought to remove the potential occurrence of GM. It should also be noted that probabilistic tractography is influenced not only by the true underlying probability of an anatomical connection, but also by factors such as the distance from a seed voxel and the geometry of the pathways being tracked with more tortuous pathways or pathways involving crossing fibers being more difficult to track [81]. Probabilistic tractography is also insensitive to the polarity of axonal projections. However, the probabilistic method employed in the current study uses multi-fibre tractography which offers significant advantages over deterministic methods when tracking difficult white matter tracts such as those that include crossing fibres [81]. It is important to note that due to the difficulties of obtaining sufficient numbers of ApoE4 carriers, it has not been possible to validate the current SVM method in an independent sample. While 10 times 10-fold cross validation was used to ensure performance generalisation, validation in an independent sample will be essential to determine how robust the current approach is when applied to a fully independent dataset. Recent work adopting the current pipeline has been successfully used to classify DTI images from older subjects into MCI and healthy older categories [72]. While these findings from an older cohort support the validity of the SVM approach outlined here, caution should nevertheless be exercised before the method is validated in an independent sample. This is currently being pursued as part of the European DTI Study in Dementia (EDSD) initiative.

Overall, the current study demonstrates the efficacy of using DTI in conjunction with SVMs to classify ApoE4 carrier and non-carrier groups. The feature selection algorithm, ReliefF, allowed us to develop tractography from key seed regions that were identified as having significantly different diffusion properties between carrier and non-carrier groups. The current results suggest that there are very subtle structural differences between healthy ApoE4 carrier and non-carrier subjects, with carriers of the ApoE4 allele showing slight reductions in the volume of white matter tracts initiated from the seed regions identified by ReliefF. The white matter network developed from these seed regions, which incorporates the posterior and anterior cingulum, the precuneous, and the prefrontal cortex, may represent a WM network that is more susceptible to structural damage over the life course of ApoE4 carriers. Longitudinal studies would be needed to assess the diffusion properties and development of these tracts over the life span of ApoE4 carriers and non-carriers.
